# Efficient Collaborative Learning in the Industrial IoT Using Federated Learning and Adaptive Weighting Based on Shapley Values

**DOI:** 10.3390/s25030969

**Published:** 2025-02-06

**Authors:** Dost Muhammad Saqib Bhatti, Mazhar Ali, Junyong Yoon, Bong Jun Choi

**Affiliations:** School of Computer Science and Engineering, Soongsil University, Seoul 06978, Republic of Korea; saqib@ssu.ac.kr (D.M.S.B.); mazhar@soongsil.ac.kr (M.A.); wnsdyd1124@soongsil.ac.kr (J.Y.)

**Keywords:** federated learning, industrial IoT, deep neural networks, distributed learning, Shapley value

## Abstract

The integration of the Industrial Internet of Things (IIoT) and federated learning (FL) can be a promising approach to achieving secure and collaborative AI-driven Industry 4.0 and beyond. FL enables the collaborative training of a global model under the supervision of a central server while ensuring that data remain localized to ensure data privacy. Subsequently, the locally trained models can be aggregated to enhance the global model training process. Nevertheless, the merging of these local models can significantly impact the efficacy of global training due to the diversity of each industry’s data. In order to enhance robustness, we propose a Shapley value-based adaptive weighting mechanism that trains the global model as a sequence of cooperative games. The client weights are adjusted based on their Shapley contributions as well as the size and variability of their local datasets in order to improve the model performance. Furthermore, we propose a quantization strategy to mitigate the computational expense of Shapley value computation. Our experiments demonstrate that our method achieves the highest accuracy compared to existing methods due to the efficient assignment of weights. Additionally, our method achieves nearly the same accuracy with significantly lower computational cost by reducing the computation overhead of Shapley value computation in each round of training.

## 1. Introduction

Industry 4.0 has emerged from the fusion of the Industrial Internet of Things (IIoT) and AI, transforming the operations paradigm of manufacturing companies to become more intelligent and efficient. This adaptation towards a robust industrial system is made possible through the integration of multiple intelligent devices, reliable communication networks, and high computational power within IIoT ecosystems [1]. Smart industrial systems utilize numerous devices, sensors, actuators, and machines, generating an immense amount of data that is impossible to analyze manually by humans [2,3]. Machine learning and AI applications address these challenges in data analysis, providing insights that facilitate timely decision making for humans.

Cutting-edge machine learning and deep learning algorithms are employed in IIoT setups to extract essential insights and patterns from various data sources. These sophisticated algorithms excel at analyzing complex patterns from large datasets across different industries. However, transferring diverse datasets from different organizations to a central location for training machine learning algorithms raises concerns about sensitive information leakage from malicious attackers or intruders [4]. In the smart industrial sector, sensors associated with machinery generate large volumes of data, which are typically stored locally at the edges. This data is often transferred to a cloud server for training machine learning models to recognize patterns, leading to a significant communication burden [5,6]. Federated learning (FL) offers an alternative solution to reduce communication costs by eliminating the need to transfer the entire data volume to the cloud server [7]. Instead, the machine learning model’s weights are shared back and forth during training. Consequently, federated learning eliminates the need for centralized learning and addresses both communication and privacy concerns [8].

Collaborative learning benefits industrial consumers by helping them understand complex patterns from diverse data sources, particularly when they have limited data samples. Learning from a diverse industrial dataset can also be helpful for improving the manufacturing outputs, as different organizations possess varying data silos. Large differences in data distribution and quality between industries add complexity to federated learning and can cause the global model to diverge from the optimal solution [9]. Federated learning faces challenges such as poor data quality, highly imbalanced classes, and heterogeneity of data between clients. To address these issues, we propose using cooperative game theory based on the Shapley value (SV) to identify industries with high-quality data partitions and encourage their participation.

The rest of the article is organized as follows: the related literature is discussed in the following subsection, which also describes the problems associated with conventional federated learning methods. In Section 2, the system model is presented. The proposed approach is detailed in Section 3. Lastly, the simulation results are discussed in Section 4, with the conclusion provided in Section 5.

### 1.1. Related Work

Federated learning has been widely adopted across various sectors, including finance, healthcare, Industry 4.0, and mobile computing, to facilitate collaboration among industries. However, it faces the challenge of data heterogeneity due to variations in statistical data distribution and class imbalance across industries. This heterogeneity leads to the issue of client drift, causing divergence from the global optimum. Several approaches, such as FedProx, Scaffold, and FedNova, have been proposed to address the industry drift issue, each based on its own theoretical assumptions [10,11,12,13]. Various practices, such as a mixture of global and local models, shared representation learning, fine-tuning global models at clients, and regularization loss at clients, have been implemented to address the non-independent and identically distributed (non-IID) nature of data across clients [14,15,16,17].

In this paper, the term client will be used to refer to the local industry proactively engaged in the federated learning system. Federated multi-task learning aims to address statistical and system challenges by tackling dual problems through the MOCHA method [18]. It also generalizes distributed optimization methods like CoCoA to address system challenges associated with network size and node heterogeneity. Similarly, another approach in multitask federated learning involves keeping shared model representations or modules at the server, which clients then use for their specified tasks, reducing communication and computational costs [19].

The Shapley value, originating from cooperative game theory, offers a rigorous method to fairly distribute the total gains or costs among players based on their individual contributions [20]. In the context of machine learning, SVs are utilized to attribute the contribution of each feature to the prediction of the model in a fair and interpretable manner. The SV has been utilized in federated learning by numerous authors. In [21], the authors proposed a Shapley value-based federated learning approach called FedSV, which assesses the significance of data samples in the client dataset that contribute to the convergence of the global model. This helps measure the relative contribution of each independent client, which is studied in the context of federated learning client selection, fairness, incentive, and reward mechanisms. However, in several Shapley-based federated studies, the computation and communication costs are concerning factors that make the SV approach impractical for the industrial application of FL. The authors in [21] have analyzed how different data sources from clients impact the overall performance of a global model using cooperative game theory. They utilized the Monte Carlo method for federated SV to study how changes in data distribution among participants within the same round affect the model generalization. They conducted comprehensive experiments on tasks including noisy label detection, adversarial client involvement, and data summarization. Similarly, the study [22] introduced Local Shapley at the client side that captures the client contribution based on the data imbalance and non-IID federated settings. A quantization approach has also been employed with the Local Shapley to dynamically aggregate the local models weights at the central server. Experimental results on CIFAR10 and MNIST show that FedSV outperformed the vanilla aggregation approaches such as FedAVG and FedSGD. The study on Shapley values aggregate CP-decomposition models (CPSV) [23] provides efficient communication by quantization of the parameter shared to the central server and dropping the clients based on the Shapley value approaches. CP-decomposition removes the unnecessary parameters in the model and SV determines the malicious clients or those who have less contribution in the convergence of the global model, thus also reducing the frequency of communication rounds by eliminating the server nodes responsible for hierarchical aggregation. Moreover, ref. [24] has proposed the Guided Truncation Gradient Shapley (GTG-Shapley) approach to reduce the computing cost of sub-model reconstruction from scratch for each evaluation round, as it uses the last gradient updates, respectively. Experimental results demonstrate the effectiveness of GTG-Shapley over the other SV-based federated contribution evaluation techniques on computation and achieving higher accuracy.

The data silo within the federation is often heterogeneous and imbalanced. Additionally, some clients introduce corrupt data and fake updates to gain maximum rewards, making federated learning untrustworthy. To tackle these issues, authors in [25] have employed an adaptive weighting method to aggregate the local updates based on the surrogate federated Shapley value. Similarly, Song T. et al. analyzed Shapley-based data valuation on a horizontal federation to fairly reward clients with good data samples [26]. They introduced a contribution index to measure each client’s contribution using two gradient-based comparison approaches, which reduces the computation cost as it saves the intermediate result during the training rounds. Furthermore, ref. [27] has discussed the application of Monte Carlo sampling to measure the contribution index and decrease the computational complexity of multiple agents on credit assignments in reinforcement learning. Similarly, ref. [28] applied the SV-based approach in federated learning to incentivize clients, ensure a fair distribution of profits, and promote interpretability in the federated ecosystem. Moreover, the adoption of federated learning in large-scale distributed systems, such as those involving hundreds or thousands of clients, may also introduces unique challenges that can significantly affect both convergence and stability. To be more specific, in large-scale systems, client datasets are typically highly non-IID, exhibiting significant variability in size, quality, and distribution. This heterogeneity poses two primary challenges. First, the divergence in data distributions among clients can result in conflicting model updates, slowing down the convergence process. Second, the over-representation of clients with larger datasets or more frequent participation can disproportionately influence the global model, introducing bias and reducing its overall generalizability.

Shapley value ensures the fair distribution of incentives by utilizing clients’ contributions based on data quality, computation capacity, and communication capability. The authors in [29] proposed a method called S-FedAvg, which explores the use of the Shapley value approach from game theory to select clients that positively impact the global learning objective while bypassing irrelevant clients during training. S-FedAvg demonstrated that even a small number of irrelevant client updates can significantly impact the convergence of the global model. Furthermore, in [30], a new aggregation scheme, ShapFed-WA, is proposed that uses the Shapley value to address the class imbalance issue in federated learning and it outperforms traditional FedAvg. Moreover, the authors in [31] highlighted the time constraints for communication in a heterogeneous federated setup in GREEDY FED. They used relative Shapley values to greedily select the clients for timely communication. Geng, K. et al. studied the possible attack and security issues in preserving fairness in federated SV [32]. They considered a Privacy-Preserving Contribution Evaluation (PPCE) method to protect the fairness of an incentive mechanism based on gradient Shapley, arithmetic sharing, and asymmetric encryption.

The Shapley value has been widely used to address challenges in federated learning, particularly for managing data heterogeneity and aggregating local models to achieve optimal performance. While previous studies primarily focused on using Shapley values for incentive mechanisms and calculating marginal contributions to reward users, these approaches often overlooked clients’ training performance. Additionally, they introduced significant computational complexity due to the numerous calculations required to determine average contributions. To the best of authors’ knowledge, conventional Shapley-based methods in federated learning calculate the marginal contribution of each client by considering all possible subsets of clients. While this approach provides an accurate measure of each client’s contribution, it suffers from exponential computational complexity, making it impractical for large-scale systems with numerous clients. These methods also focus heavily on incentive mechanisms or rewarding users based on their contributions, often without optimizing for computational efficiency or real-time applicability.

In contrast, our method introduces a more efficient approach by grouping clients based on their individual contributions and selecting representative clients from each group. Specifically, we calculate the individual contribution of the client in each training round. Clients are then organized into contribution levels, defined in ranges from 0 to 1. For each level, the client with the highest contribution is selected as a representative, thereby significantly reducing the number of computations required for Shapley value calculations. This grouping strategy ensures that contributions from clients across all accuracy ranges are represented, avoiding the need for exhaustive calculations while preserving the diversity of contributions. By assigning aggregation weights based on these representative contributions, our method achieves fair and effective aggregation without the computational burden of traditional Shapley-based methods. Additionally, this approach improves scalability, making it suitable for large-scale federated learning systems involving hundreds or thousands of clients. In summary, we have integrated the Shapley value for adaptive weighting in such a way that a promising solution is achieved. By quantifying each client’s contribution to the global model, Shapley values enable more equitable weighting during aggregation, ensuring that clients with valuable but smaller or more unique datasets are appropriately represented. This approach helps mitigate the effects of data heterogeneity, reduces the risk of over-representation by dominant clients, and fosters more stable and efficient convergence in large-scale federated learning systems.

In other words, we propose a novel application of the Shapley value that focuses on enhancing global training performance by efficiently aggregating local models. Our method achieves a balance between performance and computational efficiency, significantly reducing complexity and latency. This makes the approach particularly suitable for resource-constrained and time-sensitive IIoT environments, where traditional methods may not perform well.

### 1.2. Contributions

The main goal of this paper is to develop an effective aggregation method that successfully integrates essential parameters, including the impact of each client on global training. The contributions of the proposed approach are summarized in the following.

- An efficient global training method for industrial IoT is proposed, using cooperative game theory concepts. It assigns aggregation weights based on the Shapley value of clients, the size of their datasets, and data heterogeneity.
- The global model is refined to minimize prediction loss and improve classification accuracy. This strategy is assessed through comprehensive simulations with a federated learning simulator across different data heterogeneity scenarios, showing higher performance compared to traditional methods.
- The proposed method calculates Shapley values, quantizes contributions, and performs aggregation to achieve the highest accuracy with the lowest computational complexity. This is accomplished by accurately aggregating local models trained by industries, considering parameters such as their Shapley contributions, data heterogeneity, and data volume.

## 2. System Model

Consider a network with *K* collaborative industries, each deployed with neural networks and communicating with a server for global training. Each industry, referred to as a client, aims to update its local model using the data available to it. The server, in turn, updates the global model by aggregating the updated local models from all clients. If we assume that the *k*-th client in the network holds $D_{k}$ data comprising $n_{k}$ samples and $\kappa_{k}$ classes of labels.

**Figure 1:**
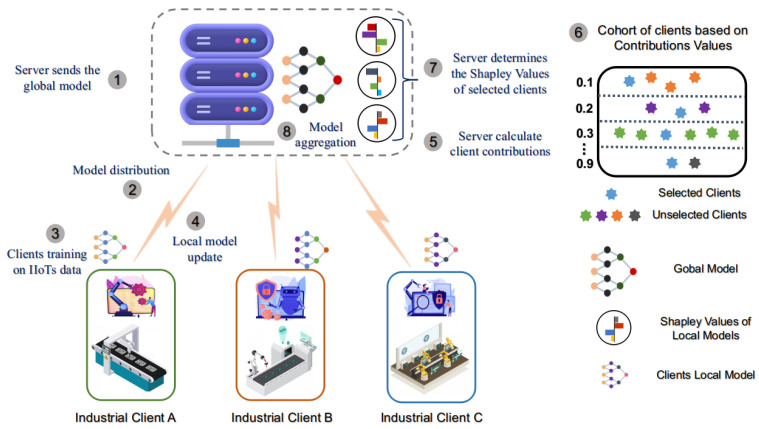
Federated learning using cooperative game theory to enhance efficiency.

Global training is initiated when the server shares the initial global model with all local industries. Each client performs local training and shares the local model weights with the server. Based on the trained local model of a client, the server calculates that client’s Shapley contribution. A group *M* is a subset of *K* that collaborates to complete a task. The utility function υ(M) (where M⊆K) represents the utility of a group *M* for a task, such as the accuracy of the central model trained with *M* group. The marginal contribution of client *k* with respect to a group *M* is given by υ(M∪{k})−υ(M). Figure 1 depicts the working principle of our approach, which starts with the model initialization at the server.

Industrial clients receive the global model from the server and train it on their data samples. Training on heterogeneous client IIoT data results in independent client models, which are sent back to the server for aggregation. The server computes the client contribution value (CV) based on the accuracy metric and forms cohorts of similar clients’ CV thresholds using the validation training samples. The server selects the most suitable client from each cohort to calculate the SV through relative permutation in a game theory mechanism. At last, the server aggregates the model based on marginal contribution, accuracy contribution, and data volume of the selected clients.

Furthermore, how Shapley contributions, along with the data volume and heterogeneity of each client, play a role in assigning the aggregation weight of each client is discussed in Section 3. The list of symbols used is given in Table 1 below.

## 3. Proposed Method

### 3.1. Local Training

The goal is to utilize industrial data to improve classification accuracy and minimize the loss function of the global model on the server. The primary objective is to achieve the lowest possible loss when predicting any given sample, $(x_{i},y_{i})\in D$, from the industrial dataset. This objective is to minimize the loss on the industry’s dataset, *D*, using the global model, ω, which can be formulated as

(1) $$\min_{\omega \in R}\;\ell \left(\omega ,D\right),$$

where $\ell \left(\omega ,D\right)=\frac{1}{n}\sum_{i=1}^{n}\ell_{i}\left(\omega ,D\right)$, with $\ell_{i}\left(\omega ,D\right)$ representing the prediction loss.

In each round *t*, the global model $\omega^{t}$ is distributed to all local industries. These industries then use their local data to calculate gradients and update the local models. The gradients for the *k*th client at round *t* is computed as

(2) $${ð}_{k}^{t}=\nabla_{\omega_{k}^{t}}\ell \left(\omega_{k}^{t},D_{k}\right),$$

where $D_{k}$ represents the dataset of the *k*th client, containing $n_{k}$ samples $\left(x_{k_{i}},y_{k_{i}}\right)$ for $1\leq i\leq n_{k}$. The local objective for this client can be formulated as

(3) $$\min_{\omega_{k}^{t}\in R}\;\ell \left(\omega_{k}^{t},D_{k}\right),$$

where $\ell (\omega_{k}^{t},D_{k})$ is the prediction loss given by

(4) $$\ell \left(\omega_{k}^{t},D_{k}\right)=\frac{1}{n_{k}}\sum_{\left(x_{k_{i}},y_{k_{i}}\right)\in D_{k}}f_{k_{i}}\left(\omega_{k}^{t}\right).$$

The loss function $f_{k_{i}}\left(\omega_{k}^{t}\right)$ for predicting the given samples $\left(x_{k_{i}},y_{k_{i}}\right)$ using the model $\omega_{k}^{t}$ is defined as $L(\omega_{k}^{t},x_{k_{i}},y_{k_{i}})$. The above Equation (2) can be rewritten as

(5) $${ð}_{k}^{t}=\nabla_{\omega_{k}^{t}}\sum_{\left(x_{k_{i}},y_{k_{i}}\right)\in D_{k}}L\left(\omega_{k}^{t};x_{k_{i}},y_{k_{i}}\right).$$

Once the gradients are calculated, the *k*th client’s local model is updated, which can be expressed as

(6) $$\omega_{k}^{t+1}\leftarrow \omega_{k}^{t}-\eta_{k}{ð}_{k}^{t},$$

where $\eta_{k}$ is the learning rate for the *k*th client. The updated local model for that client at round t+1 is $\omega_{k}^{t+1}$, which is then sent back to the server. Furthermore, the accuracy for the *k*-th client in a given training round *t* is calculated as

(7) $${\mathrm{Accuracy}}_{k}^{t}=\frac{\mathrm{Number}\;\mathrm{of}\;\mathrm{correctly}\;\mathrm{predicted}\;\mathrm{samples}}{n_{k}}\times 100$$

where ${\mathrm{Accuracy}}_{k}^{t}$ represents the percentage of correctly classified samples on the local validation dataset of the *k*-th client in round *t*. This metric evaluates the performance of the client’s model during local training.

### 3.2. Shapley Contribution

Once the local models have been trained, they are sent to the server, where the server evaluates the individual contribution of each industry. In order to improve the performance of global training, we have computed the Shapley value for each client to measure its marginal contribution to the global model. These values are then used as weights to aggregate local model updates. The Shapley value for a client k is defined as the average marginal contribution of that client across all possible subsets of the other clients. Mathematically, for a set of *K* clients, the Shapley value $\phi_{k}\left(\upsilon \right)$ for client *k* at round *t* is given as

(8) $$\phi_{k}^{t}\left(\upsilon \right)=\sum_{M^{t}\subseteq K\setminus \left\{k\right\}}\frac{|M^{t}\left|!\;(|K|-\right|M^{t}\left|-1\right)!}{|K|!}\;\left[\upsilon \left(M^{t}\cup \left\{k\right\}\right)-\upsilon \left(M^{t}\right)\right],$$

where *K* represents the set of all clients. $M^{t}$ denotes a subset of *K* that does not include client *k*. Moreover, $|M^{t}|$ is the cardinality of a subset $M^{t}$. Additionally, $\upsilon (M^{t})$ is the value function, such as the model’s prediction for the subset *M*. Furthermore, $\left(\upsilon \left(M^{t}\cup k\right)\right)$ is the value function for the subset *M* with client *k* added.

To reduce complexity, we have grouped clients into levels based on their individual contributions $c_{k}^{t}$ and select a representative client from each level. First, calculate the individual contribution $c_{k}^{t}$ of *k*-th client in round *t*, which is referred to as the accuracy of that client in that specific round. Afterwards, we have defined levels of accuracy from 0 to 1 with a step size of σ, then select the highest-contributing feature within each level. In other words, if we assume that σ is 0.1, then, for instance, Level 0 includes clients’ features with contributions ranging from 0.0 to 0.1. Level 1 includes features with contributions ranging from 0.1 to 0.2. This pattern continues incrementally up to Level 9, which includes features with contributions ranging from 0.9 to 1.0. Assuming that accuracy is divided into $L_{j}$ levels, it can be written as

(9) $$L_{j}=\left\{k\in K|j\leq c_{k}<j+\sigma \right\}\;\;\;\mathrm{for}\;j=\left\{0,\sigma ,2\sigma ,\ldots ,1-\sigma \right\}.$$

For each level $L_{j}$, we have selected the client $k_{j}^{t}$ with highest contribution as

(10) $$k_{j}^{t}=argmax_{k\in L_{j}}c_{k}^{t}.$$

This approach allows us to consider a smaller set of representative features, reducing the computational burden while maintaining meaningful contributions. Hence, the proposed equation for calculating the Shapley contribution for $k_{j}^{t}$-th client of *j*-th level at round *t* is denoted as

(11) $$\begin{array}{c} \phi_{k_{j}^{t}}^{t}\left(\upsilon \right)=\sum_{M^{t}\subseteq \hat{K^{t}}\setminus \left\{k_{j}^{t}\right\}}\frac{|M^{t}\left|!\;(\right|\hat{K^{t}}\left|-\right|M^{t}\left|-1\right)!}{|\hat{K^{t}}|!}\;\;\;\;\;\;\;\;\;\;\;\\ \;[\upsilon \left(M^{t}\cup \left\{k_{j}^{t}\right\}\right)-\upsilon \left(M^{t}\right)]. \end{array}$$

Our approach calculates the Shapley value for the *k*-th feature by first selecting representative features from each level based on their contributions. These representative features are then used to form a smaller subset $\hat{K}$. The Shapley value for the *k*-th client is computed using $\hat{K}$, which reduces computational complexity while still maintaining significant client contributions. This marginal contribution is further utilized in global model training, resulting in enhanced performance. In other words, the Shapley value calculated for each client evaluates its marginal contribution to the global model. These values are then used as weights for aggregating local model updates, enhancing accuracy by assigning aggregation weightage according to each client’s true contribution, resulting in a more balanced and effective global model.

### 3.3. Global Model Training

Upon receiving the locally trained models, the server calculates the individual contribution of each client based on accuracy and segregates the clients as defined contributions levels. After selecting the range of clients based on their individual contribution, the Shapley marginal contribution is calculated, which is used for assigning the aggregation weightage in global training. The global model is updated as

(12) $$\begin{array}{c} \omega^{t+1}=\sum_{k_{j}^{t}}^{K^{t}}[\left(1-\gamma -\beta \right)\left(\frac{\phi_{k_{j}}^{t}\left(\upsilon \right)}{\phi^{t}\left(\upsilon \right)}\right)+\left(1-\alpha -\gamma \right)\\ \;\;\;\;\;\;\;\;\;\;\;\;\;\;\;\;\;\;\;\;\;\left(\frac{\kappa_{k_{j}}}{\kappa^{t}}\right)+\left(1-\alpha -\beta \right)\left(\frac{n_{k_{j}}}{n^{t}}\right)]\omega_{k_{j}}^{t+1}, \end{array}$$

where $\phi_{k_{j}}^{t}\left(\upsilon \right)$, $\kappa_{k_{j}}$, and $n_{k_{j}}$ represent the marginal contribution, classes of labels, and data volume of the *k*-th client, respectively. The terms $\phi^{t}\left(\upsilon \right)$, $\kappa^{t}$, and $n^{t}$ are defined as follows: $\phi^{t}\left(\upsilon \right)=\sum_{k_{j}}^{\hat{K}}\phi_{k_{j}}^{t}\left(\upsilon \right)$, $\kappa^{t}=\sum_{k_{j}}^{\hat{K}}\kappa_{k_{j}}$, and $n^{t}=\sum_{k_{j}}^{\hat{K}}n_{k_{j}}$. Furthermore, (1−γ−β) represents the weight assigned to the marginal contribution of *k*-th industry, (1−α−γ) denotes the aggregation weight allocated to data label classes, and (1−α−β) indicates the aggregation weight attributed to the number of samples on which k-th client trained the local model.

The Equation (12) integrates weighted contributions from various data characteristics, with weights (α,β,γ) representing the relative importance of marginal contribution, data heterogeneity, and data volume in the aggregation process. These weights provide the flexibility to adjust the emphasis on different data characteristics according to the specific requirements or priorities of the learning task. In addition to that, the equation evaluates the aggregated metrics ($\phi_{k_{j}}^{t}\left(\upsilon \right)$, $\kappa_{k_{j}}$, and $n_{k}$), which represent the marginal contribution of the industry, the classes of labels in that industry’s data, and data volume, respectively. These aggregated metrics provide a comprehensive overview of the characteristics of the collective data characteristics across the group of industries, facilitating more informed decision-making during the aggregation process. To be more specific, the parameter α represents the weight assigned to the Shapley contribution of each industry, ensuring fair aggregation by accounting for the industry’s actual impact on the global model. This weighting mechanism allows the contributions of industries to be evaluated more equitably, especially in scenarios where the significance of their data goes beyond its sheer volume. Given the critical role of the Shapley contribution in determining each industry’s current status and relevance in the training process, α was set to 0.5 to emphasize its importance in the aggregation process. In contrast, β denotes the weight assigned to the volume of the industry’s data, helping balance the influence of clients with larger datasets. This ensures that industries with extensive data do not disproportionately dominate the global model while still recognizing the value of their contributions. Finally, γ captures the weight given to the variation within an industry’s data, addressing the significance of data diversity in federated learning. By considering data variability, the global model benefits from the inclusion of diverse patterns, improving generalizability and robustness. The choice of α=0.5, β=0.25, and γ=0.25 reflects an empirically determined balance, with a higher priority given to Shapley contributions due to their role in dynamically assessing the significance of each client’s input. This ensures a well-rounded aggregation that enhances both fairness and performance.

Our proposed algorithm is described in Algorithm 1.

The algorithm begins by iterating through several rounds of training, denoted by *T*, and each client *k* in the set of clients *K* initializes its local model training, as mentioned in lines 1–3. For each client *k*, the local model parameters are updated using the gradient descent method. Specifically, $\omega_{k}$ represents the local model parameter for client *k*, $\eta_{k}$ is the learning rate of *k*-th client, and ${ð}_{k}^{t}$ is the gradient, as described in lines 4–8. The individual contribution of each client *k* is calculated and stored as $c_{k}$, as per lines 9–12. Contribution levels are defined based on these calculated contribution values. Clients are selected from each level based on their contribution values falling within predefined intervals and are grouped for calculating the marginal contributions. For each level $L_{j}$, a representative client $k_{j}$ is selected. This selection is based on specific criteria, such as having the highest or most representative contribution value at that level, as mentioned in lines 13–19. For each client $k_{j}$ in the set of selected clients $\hat{K^{t}}$, the Shapley value is computed. The Shapley value is calculated considering all possible subsets *M* of clients excluding $k_{j}$, determining the marginal contribution of adding $k_{j}$ to each subset. The marginal contribution is then averaged over all subsets to obtain the Shapley value, which reflects the importance of each client’s contribution, as detailed in lines 20–25. Once the marginal contribution is calculated, the global model is updated based on our proposed equation, as mentioned in lines 26–28. Our contribution lies in reducing the complexity of the Shapley value calculation in lines 13–19 and the aggregation weightage assigned in global model training in lines 26–29.
**Algorithm 1** Proposed method for global training 1:**for** roundt=0,1,2,…,T **do** 2:   **Initialization of local training**   ∀clientsk=1,2,…,K. 3:   **for** eachclientk∈K **do** 4:     $\omega_{k}^{t+1}\leftarrow \omega_{k}^{t}-\eta_{k}{ð}_{k}^{t}$ 5:   **end for**  6:   **Calculate Individual Contributions** 7:   **for** each feature *k* in *K* **do** 8:     $c_{k}\leftarrow$ calculate contribution of (*k*) 9:   **end for**10:   **Define Levels Based on Contribution Values**11:   **for** j=0,σ,2σ,…,1−σ **do**12:     $L_{j}=\left\{k\in K|j\leq c_{k}<j+\sigma \right\}\;$13:   **end for**14:   **Select Representative Clients from Each Level**15:   **for** levels $L_{1},L_{2},\ldots L_{j}$ **do**16:     $k_{j}^{t}=argmax_{k\in L_{j}}c_{k}^{t}$17:     $\hat{K^{t}}\leftarrow k_{j}^{t}$18:   **end for**19:   **Compute Shapley Value using Selected Clients**20:   **for** each $k_{j}^{t}$ in $\hat{K^{t}}$ **do**21:     **for** each subset $M^{t}\subseteq \hat{K^{t}}\setminus \left\{k_{j}^{t}\right\}$ **do**22:        Shapley Weight $\leftarrow \frac{|M^{t}\left|!\;(\right|\hat{K^{t}}\left|-\right|M^{t}\left|-1\right)!}{|K^{t}|!}$23:        Marginal contribution $\leftarrow \upsilon \left(M^{t}\cup \left\{k_{j}^{t}\right\}\right)-\upsilon \left(M^{t}\right)$24:        $\phi_{k_{j}}^{t}\left(\upsilon \right)=\sum_{M^{t}\subseteq \hat{K^{t}}\setminus \left\{k_{j}^{t}\right\}}\frac{|M^{t}\left|!\;(\right|\hat{K^{t}}\left|-\right|M^{t}\left|-1\right)!}{|\hat{K^{t}}|!}\left[\upsilon \left(M^{t}\cup \left\{k_{j}^{t}\right\}\right)-\upsilon \left(M^{t}\right)\right]$25:     **end for**26:   **end for**27:   **Global Model Training**28:   $\omega^{t+1}=\sum_{k_{j}^{t}}^{K^{t}}[\left(1-\gamma -\beta \right)\left(\frac{\phi_{k_{j}}^{t}\left(\upsilon \right)}{\phi^{t}\left(\upsilon \right)}\right)+\left(1-\alpha -\gamma \right)$29:   $\;\;\;\;\;\;\;\;\;\;\;\;\;\;\;\;\;\;\;\;\;\left(\frac{\kappa_{k_{j}}}{\kappa^{t}}\right)+\left(1-\alpha -\beta \right)\left(\frac{n_{k_{j}}}{n^{t}}\right)]\omega_{k_{j}}^{t+1}$30:**end for**

The above Equation (12) encapsulates an advanced aggregation process aimed at enhancing global training in federated learning. It represents the combination of updates from individual industries, leveraging distributed data while ensuring privacy and security. At its core, the equation computes the aggregated result by utilizing the marginal contribution of each industry. Each industry’s contribution to the aggregation process is based on its unique data characteristics, including the marginal contribution, data volume, and data label classes.

## 4. Experiments

In our simulation, a number of tests were performed to verify that the proposed method improved the performance. The results shown in the simulation section are the average of all conducted tests. The experiments were conducted on a system equipped with an NVIDIA RTX A6000 GPU. The implementation was carried out using Python 3.8 with PyTorch 1.10 for model training and simulation of federated learning.

To simulate a network of 10 randomly distributed clients, the dataset was partitioned with various non-IID distributions. Each client was randomly assigned a percentage of the dataset, varying in size from 5% to 15%, to replicate real-world data heterogeneity.

For scalability analysis, the number of clients was increased to 20 and 50, while maintaining the same non-IID data distribution strategy. The results indicate that as the number of clients increases, the computational complexity rises. However, the proposed grouping strategy significantly mitigates the impact of these complexities, ensuring stable performance and convergence. Performance metrics such as accuracy, precision, recall, and F1 score remained consistent across different client scales, with slight variations due to increased data heterogeneity. These findings demonstrate the scalability and robustness of the proposed method in large-scale federated learning scenarios.

The integer labels of the data are encoded using one hot encoder, which creates a binary column for each label and returns a dense array. The clients are randomly located in a geographical area and the training data is randomly distributed among *K* number of clients under Non-IID manner. Some clients may have very limited data and others may have numerous data samples. The proposed method along with conventional methods is implemented using TensorFlow 2.2.0. Each client updates the local model with a batch size of 32 and 1 epoch per communication round.

**Figure 2:**
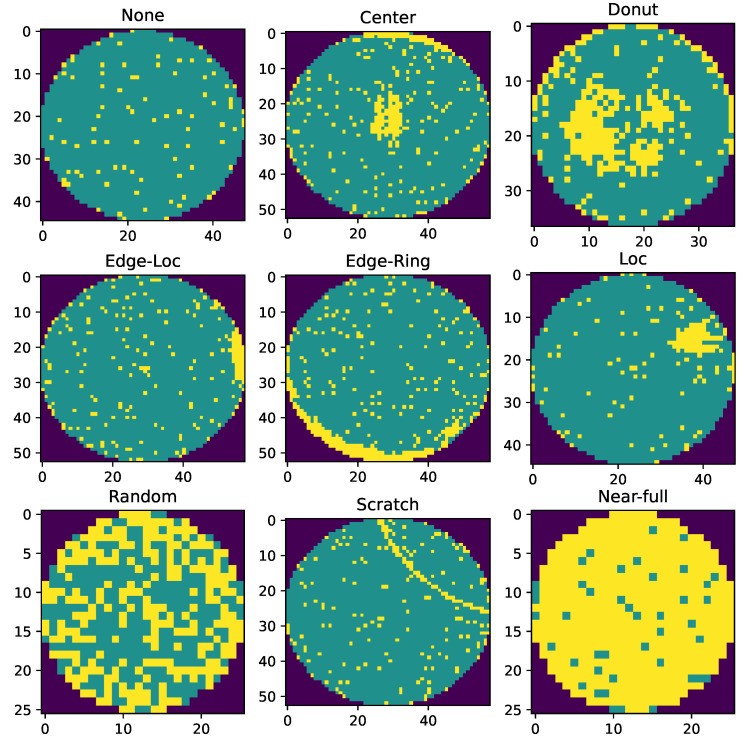
Wafer manufacturing issues.

The client’s learning model for the classification problem is the CNN model with a Conv2D layer having 64 filters with 3×3 filter size and ‘Relu’ activation function, one hidden layer of 200 neurons with the same activation function, and one output layer with ‘Softmax’ function. The clients’ loss is calculated using the categorical cross-entropy. The model trained by the client is employed with SGD having a learning rate of $\eta_{k}=0.01$. For the object detection problem, we have used YOLO v8. For the classification problem, we used the Silicon wafers dataset [33], which includes six classes: center, donut, edge-loc, edge-Ring, loc, random, scratch, near-full, and none as shown in Figure 2. The yellow regions in each class’s image represent wafer defects.

These issues arise during the manufacturing of silicon. For the object detection problem, we utilized the printer circuit board (PCB) dataset [34] to identify defects such as missing holes, mouse bites, open circuits, shorts, spurs, and spurious copper.

Furthermore, we have implemented and compared our method using a 3D convolutional neural network with a jester dataset [35], focusing on distinguishing between multiple hand gesture categories. The objective was to perform action recognition on the Jester dataset, focusing on distinguishing between multiple hand gesture categories. Specifically, we have used inputs of shape (3, 30, 128, 128), corresponding to 30 frames of 128 × 128 resolution with 3 color channels. The eventual goal of our proposed method is to acquire improved performance.

We have compared our method with several state-of-the-art federated learning approaches that use Shapley values, including AfedSV+ [25], Shap Federated [36], FedSV [21], and S-FedAvg [29]. Shap Federated uses the conventional method of Shapley value calculations. FedSV is an algorithm extended for robust federated learning using a variant of the Shapley value. AfedSV+ is a modified version of FedSV. S-FedAvg is an algorithm that addresses irrelevant data or clients by modifying FedAvg and selecting relevant clients based on an SV-based score.

**Figure 3:**
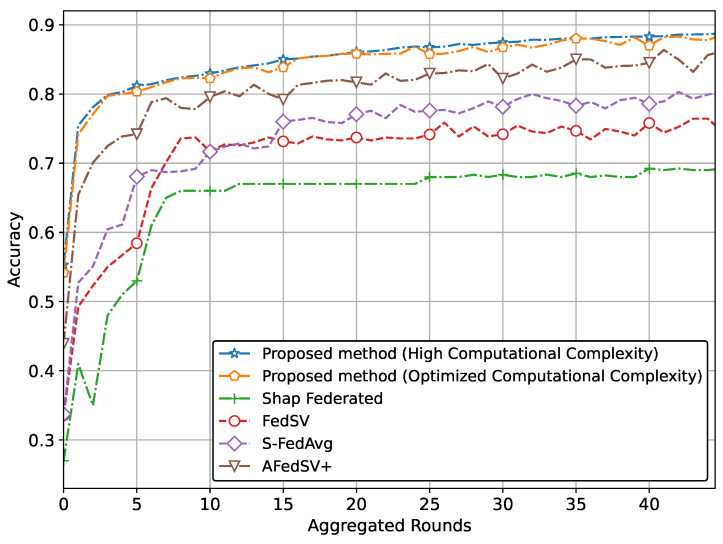
Accuracy comparison of the proposed method with classification problem on silicon wafers data.

**Figure 4:**
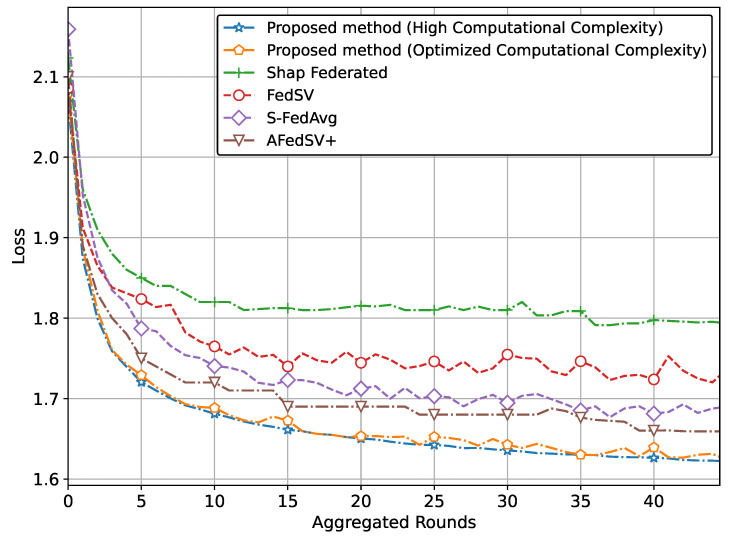
Loss comparison of proposed method with classification problem on silicon wafers data.

The performance of the proposed methods for the classification problem on silicon wafer data, showing superior accuracy and better convergence behavior compared to other methods is shown in Figure 3 and Figure 4.

Specifically, Figure 3 illustrates the superior accuracy of the proposed method compared to conventional methods. We have presented two versions of the proposed method. The version with the highest accuracy incorporates Shapley values and achieves this superior accuracy due to our proposed global aggregation equation. The second version, which reduces computational complexity by involving a limited calculation of Shapley values, also achieves almost similar accuracy to the high computational complexity version. Figure 4 also clearly shows that the proposed method, particularly the version utilizing Shapley values and the global aggregation equation, achieves the lowest loss values, demonstrating superior performance. The optimized computational complexity version also performs exceptionally well, almost mirroring the high-complexity version, thereby validating the effectiveness of our optimization. Conventional methods, while showing some reduction in loss, do not reach the same level of performance as our proposed methods.

**Figure 5:**
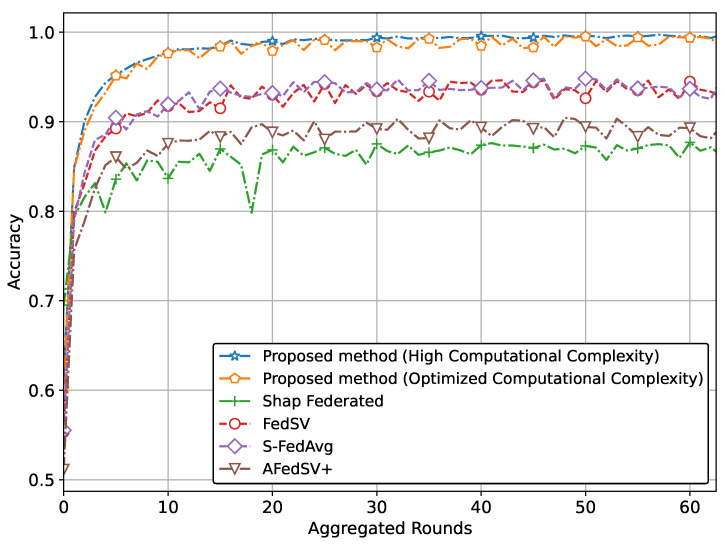
Accuracy comparison of the proposed method for gesture recognition.

Moreover, we have compared our method using a 3D convolutional network for gesture recognition. Figure 5 depicts the accuracy trends of the proposed method alongside conventional methods. The results demonstrate that the proposed method with high computational complexity achieves the highest accuracy among all approaches, showcasing its effectiveness in aggregating diverse client contributions. When the computational complexity is optimized, the accuracy of the proposed method slightly decreases but remains comparable to the high-complexity version. This indicates that optimizing computational complexity does not significantly compromise performance. In contrast, the conventional methods consistently show lower accuracy, further emphasizing the advantages of the proposed approach.

**Figure 6:**
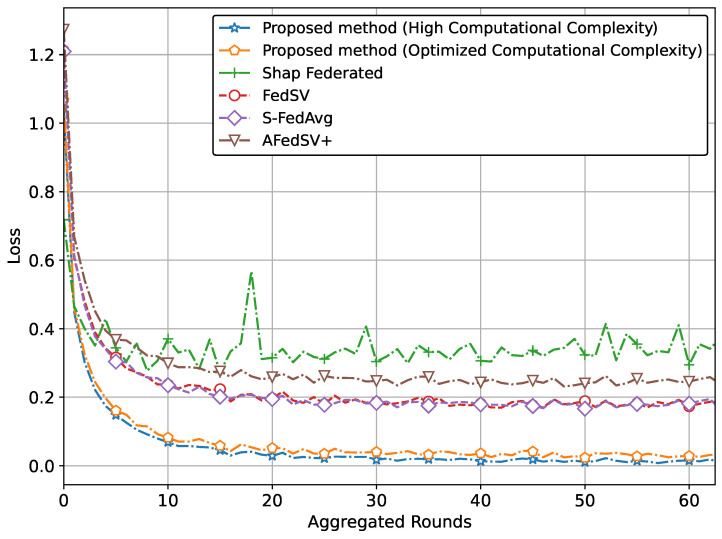
Loss comparison of the proposed method for gesture recognition.

Furthermore, Figure 6 presents the loss trends for the proposed method and conventional methods. The proposed method with high computational complexity achieves the lowest loss, indicating superior convergence and model optimization. When the computational complexity is optimized, the loss slightly increases but remains close to that of the high-complexity version, highlighting the method’s ability to balance performance with efficiency. Conventional methods exhibit consistently higher loss values, reinforcing the effectiveness of the proposed method in achieving better convergence and model quality, even under optimized complexity.

**Figure 7:**
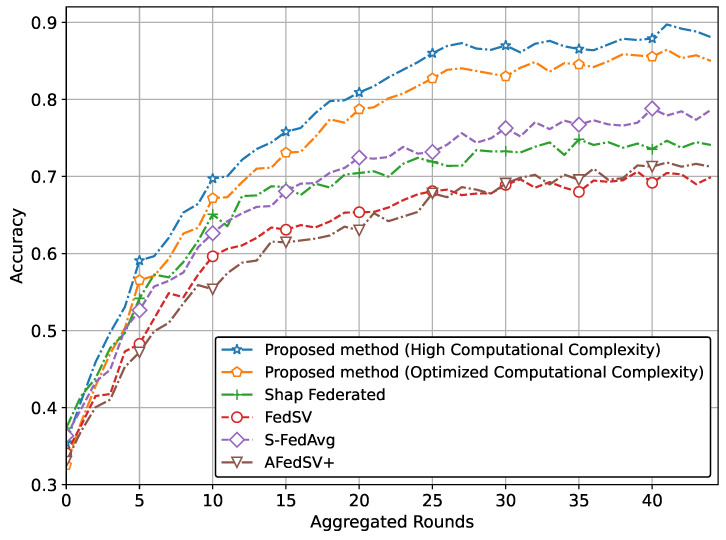
Accuracy comparison of the proposed method with object detection on PCB data.

In addition, Figure 7 illustrates the accuracy comparison between the proposed method and conventional methods for the object detection problem in PCB data.

**Figure 8:**
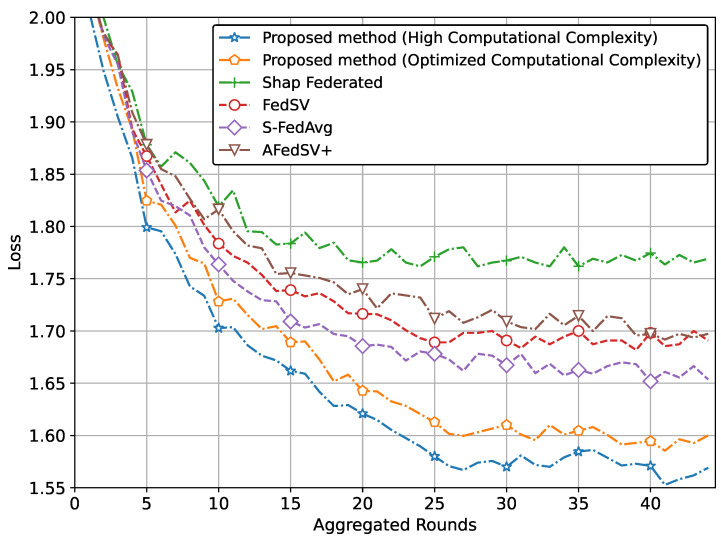
Loss comparison of the proposed method with object detection on PCB data.

It clearly demonstrates that the proposed method achieves the highest accuracy in object detection also on PCB data. The version with optimized computational complexity performs almost as well as the high-complexity version, showcasing the effectiveness of the optimization. Although conventional methods show improvements in accuracy, they do not reach the same performance levels as the proposed methods. Moreover, Figure 8 illustrates the loss comparison between the proposed method and the conventional methods.

It clearly shows that the proposed methods achieve superior performance compared to the conventional ones. The version with optimized computational complexity performs almost as well as the high-complexity version.

Furthermore, we have added a table that shows the superior performance of the proposed method on the PCB dataset compared to conventional methods in various performance metrics, including precision, precision, recall, and F1 score. The results clearly highlight that the proposed algorithm outperforms traditional approaches in all evaluated metrics, showcasing its effectiveness in addressing challenges such as data heterogeneity and computational efficiency. This comprehensive comparison given in Table 2 underscores the robustness and adaptability of our method.

**Figure 9:**
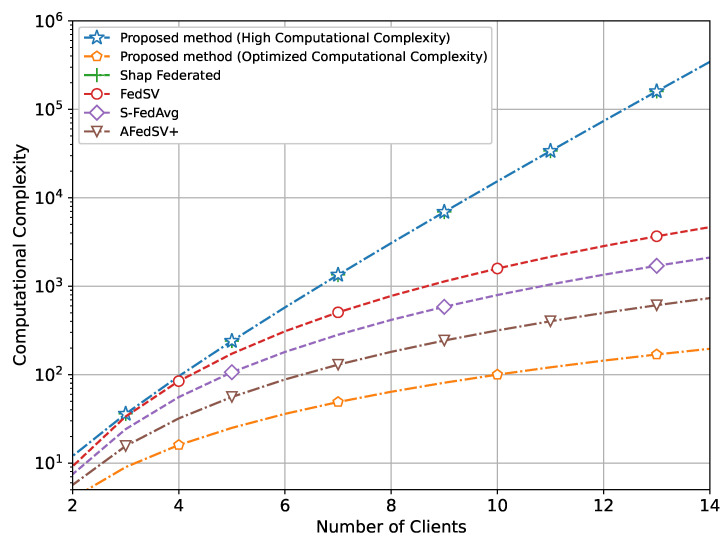
Comparison of complexity of proposed method with conventional methods.

In addition, we have also compared the computational complexity between the proposed methods and conventional methods as a function of the number of clients as shown in Figure 9.

It clearly shows that while the proposed method with high computational complexity along with Shap federated demands significant computational resources, the optimized version effectively reduces this burden. The optimized computational complexity version maintains a much lower complexity level, comparable to some conventional methods that have also made amendments in calculating the Shapley value. Our proposed method operates with significantly reduced complexity while achieving the highest training performance. This balance of efficiency and effectiveness highlights the advantages of the optimized proposed method over both the high-complexity version and conventional methods.

To summarize, the proposed method achieves notably improved performance with significantly reduced complexity. Though the study shows better performance than the relevant Shapley-based approaches, but still vulnerable to privacy breaches and susceptible to malicious attack in federated setup [37]. Smart contracts and consensus algorithms in peer-to-peer blockchain are considered to provide a better platform for federated learning prone to privacy leakage, incentive mechanisms, poison attacks, and insecure communication [38]. In future, we are interested in exploring the field of blockchain to integrate it with our Shapley-based approach to secure the communication channels and incentivize the clients in the decentralized federated learning perspective.

## 5. Conclusions

This paper proposes a method that effectively addresses non-IID data challenges and improves Industrial IoT by utilizing federated learning with Shapley values. We proposed two strategies to improve accuracy and reduce complexity. To enhance accuracy, we frame global training as cooperative games, adjusting client weights based on Shapley contributions and local dataset characteristics. To lower the computational cost of calculating Shapley values, we implement a quantization strategy, minimizing overhead by quantizing collaborative contributions in each training round.

## Figures and Tables

**Table 1:** List of symbols used in the study.

Symbol	Description
$D_{k}$	Client *k*th data samples
ω	Global model weight
${ð}_{k}^{t}$	Gradient of client *k*-th at round *t*
$\phi_{k}\left(\upsilon \right)$	Shapley value of client *k* at round *t*
*K*	Set of all clients
$M^{t}\subseteq \hat{K^{t}}\setminus \left\{k_{j}^{t}\right\}$	Subset of *K* that does not include client *k* at round *t*
$\upsilon (M^{t})$	Value function of the subset *M* at round *t*
$k_{j}^{t}$	Highest contribution client in level *j* at round *t*
$\kappa_{k_{j}}$	Class labels in *k*-th client data of level *j*
$n_{k_{j}}$	Number of samples in *k*-th client of level *j*
$\omega_{k_{j}}^{t+1}$	Local model weights of *k*-th client of level *j* at round t+1
$\hat{K^{t}}$	Subset of selected clients to compute Shapley Value at round *t*

**Table 2:** Comparison of Proposed and Conventional Methods Across Performance Metrics.

Methods	Accuracy	Precision	Recall	F-1
Proposed method (High Computational Complexity)	0.901	0.889	0.891	0.889
Proposed method (Optimized Computational Complexity)	0.878	0.868	0.871	0.869
Shap Federated	0.748	0.734	0.721	0.727
FedSV	0.701	0.686	0.690	0.687
S-FedAvg	0.788	0.775	0.771	0.772
AFedSV+	0.722	0.709	0.711	0.709

## Data Availability

Data are contained within this article.

## Abbreviations

The following abbreviations are used in this manuscript:

IIoT	Industrial Internet of Things
FL	Federated Learning
SV	Shapley Value
Non-IID	Non-Independent and Identically Distributed
GTG-Shapley	Guided Truncation Gradient Shapley
PPCE	Privacy-Preserving Contribution Evaluation
CV	Contribution Value
PCB	Printer Circuit Board
